# Supplementary material for: Comparison between the Smart Triage model and the Emergency Triage Assessment and Treatment guidelines in triaging children presenting to the emergency departments of two public hospitals in Kenya
Source: PLOS Digit Health. 2024 Aug 1;3(8):e0000408. doi: 10.1371/journal.pdig.0000408 (PMC11293692; doi:10.1371/journal.pdig.0000408)
Supplement: S1 Table — (DOCX) [file pdig.0000408.s001.docx]

**S1 Table: Distribution of participants and outcomes by triage system.**

| **Triage system** | **ETAT** | | | **Smart Triage only model** | | | **Smart Triage model**  **with independent triggers** | | | **Recalibrated Smart Triage model with independent triggers** | | |
| --- | --- | --- | --- | --- | --- | --- | --- | --- | --- | --- | --- | --- |
|  | **Emergency** | **Priority** | **Non-urgent** | **Emergency** | **Priority** | **Non-urgent** | **Emergency** | **Priority** | **Non-urgent** | **Emergency** | **Priority** | **Non-urgent** |
| Participants  n (%) | 513  (9.2) | 2003  (35.7) | 3089 (55.1) | 790  (14.1) | 2373 (42.3) | 2442 (43.6) | 1163  (20.8) | 2345 (41.8) | 2097 (37.4) | 1161  (20.7) | 1827 (32.6) | 2617  (46.7) |
| Admission distribution  n (%) | 191(37.2) | 168(8.4) | 36(1.2) | 224(28.4) | 134(5.6) | 37(1.5) | 296(25.5) | 83(3.5) | 16(0.8) | 296(25.5) | 74(4.1) | 25(1.0) |
| Mortality on the day of enrolment  n (%) | 4(0.7) | 2(0.1) | 0(0) | 4(0.5) | 2(0.1) | 0(0) | 5(0.4) | 1(0) | 0(0) | 5(0.4) | 1(0) | 0(0) |
| In-hospital mortality  n (%) | 13(2.5) | 8(0.4) | 0(0) | 14(1.8) | 5(0.2) | 2(0.1) | 18(1.5) | 3(0.1) | 0(0) | 18(1.6) | 3(0.2) | 0(0) |
| Mortality during follow-up  n (%) | 8(1.6) | 12(0.6) | 2(0.1) | 7(0.9) | 14(0.6) | 1(0) | 16(1.4) | 6(0.3) | 0(0) | 16(1.4) | 6(0.3) | 0(0) |
